# Clinical characteristics, injury pattern and management of pediatric pelvic fracture: An observational retrospective study from a level I trauma center

**DOI:** 10.1186/s12891-021-04448-6

**Published:** 2021-07-16

**Authors:** Abduljabbar Alhammoud, Isam Moghamis, Husham Abdelrahman, Syed Imran Ghouri, Mohammad Asim, Elhadi Babikir, Hassan Al-Thani, Ayman El-Menyar

**Affiliations:** 1 Department of Surgery, Orthopedic Surgery, Hamad General Hospital, Doha, Qatar; 2 Department of Surgery, Trauma Surgery, Hamad General Hospital, Doha, Qatar; 3 Clinical Research, Trauma & Vascular Surgery, Department of Surgery, Hamad General Hospital, HMC, Doha, Qatar; 4 Department of Clinical Medicine, Weill Cornell Medical School, Doha, Qatar

**Keywords:** Pediatric pelvic fracture, Fracture classification, Pelvic ring injury, Polytrauma

## Abstract

**Background:**

Pediatric pelvic fractures (PPF) are uncommon among children requiring hospitalization after blunt trauma. The present study explored our experience for the prevalence, patients demographics, clinical characteristics, injury pattern and management of pediatric pelvic fractures in a level I trauma center.

**Methods:**

This is a retrospective review of prospectively collected data obtained from trauma registry database for all pediatrics trauma patients of age ≤18 years. Data were analyzed according to different aspects relevant to the clinical applications such as Torode classification for pelvic ring fracture (Type I–IV), open versus closed triradiate cartilage, and surgical versus non-surgical management.

**Results:**

During the study period (3 and half years), a total of 119 PPF cases were admitted at the trauma center (11% of total pediatric admissions); the majority had pelvic ring fractures (91.6%) and 8.4% had an acetabular fracture. The mean age of patients was 11.5 ± 5.7, and the majority were males (78.2%). One hundred and four fractures were classified as type I (5.8%), type II (13.5%), type III (68.3%) and type IV (12.5%). Patients in the surgical group were more likely to have higher pelvis AIS (*p* = 0.001), type IV fractures, acetabular fractures and closed triradiate cartilage as compared to the conservative group. Type III fractures and open triradiate cartilage were significantly higher in the conservative group (*p* < 0.05). Patients with closed triradiate cartilage frequently sustained spine, head injuries, acetabular fracture and had higher mean ISS and pelvis AIS (*p* < 0.01) than the open group. However, the rate of in-hospital complications and mortality were comparable among different groups. The overall mortality rate was 2.5%.

**Conclusion:**

PPF are uncommon and mainly caused by high-impact trauma associated with multisystem injuries. The majority of PPF are stable, despite the underlying high-energy mechanism. Management of PPF depends on the severity of fracture as patients with higher grade fractures require surgical intervention. Furthermore, larger prospective study is needed to understand the age-related pattern and management of PPF.

## Introduction

Pediatric pelvic fractures (PPF) are uncommon with the reported incidence ranges between 0.3 and 4.0% among children required hospitalization after blunt trauma [1, 2]. This frequency of pelvic fractures is relatively lower in pediatrics as compared to adults which could be attributed to the existence of anatomical differences between the two populations [3]. The skeletally immature pediatric pelvis has several physical properties which makes it distinct from adults with respect to the injury patterns, management, and outcomes [4]. The differences include the presence of larger cartilage component in the immature bone with increased porosity of the cortical bone, greater elasticity of the pubic symphysis and the sacroiliac joint [5–7].

High-energy blunt trauma is the leading mechanism of pediatric pelvic fractures primarily due to motor vehicle accidents and fall from height [2–4]. Pelvic fracture in pediatric population is a marker of the injury severity which showed association with injuries to the head and intra-abdominal region and greater risk of hemorrhage [2, 8, 9]. Hemorrhage as reported in adult pelvic fractures that leads to hemodynamic instability is rarely observed in pediatric pelvic fracture [2, 10]. Usually solid organ injuries resulted from high-energy trauma are mostly the primary source of bleeding in children [1, 2, 11]. The adults have higher rate of mortality secondary to the pelvic fracture than the pediatric patients, in which death occurs primarily due to injuries to other body regions particularly the head [1, 8, 12].

Determination of the pattern and complexity of pediatric pelvic fracture is key for optimum management [13]. Closure of triradiate cartilage has been suggested as a distinguishing biological factor between the pediatric and adult pelvis. This factor makes the role of CT scan extremely essential for the diagnosis and management of PPF [14, 15].

Notably, simple pelvic ring injuries are the predominant type of fracture among children. Usually, PPF does not require surgical intervention as they are mostly non-displaced fractures. However, rare fractures involving the triradiate cartilage or the acetabulum as well as significant displaced fracture may require surgical management [16]. To date, there is limited literature available regarding the prevalence and outcome of pediatric pelvic fractures in the Arab Middle Eastern region. The present study explores a 3 and half year experience for pediatric pelvic fractures in a level I trauma center.

## Methods

This is a retrospective review of prospectively collected data obtained from trauma registry database at Hamad General Hospital (HGH) which has a designated as the only level 1 trauma center in the country. The trauma registry has regular internal and external validation and linked to the National Trauma Data Bank (NTDB) in the USA. Data were collected for all pediatrics polytrauma patients of age less than or equal to 18 years who were presented and treated at HGH between January 2013 and June 2016.

Variables collected and analyzed included demographic data (age, gender, nationality), mechanism of injury details, associated injuries, Glasgow Coma Score (GCS) at emergency department (ED), Injury Severity Score (ISS), Revised Trauma Score (RTS), Abbreviated Injury scores (AIS), initial vital signs, ED disposition, Focused assessment with sonography for trauma (FAST), need for intubation and blood transfusion, number of blood units transfused, massive transfusion protocol (MTP), Torode fracture classification, acetabular fracture, triradiate cartilage (open or closed), management (conservative, surgical), in-hospital complications such as pneumonia, sepsis, Acute Respiratory Distress Syndrome (ARDS), Acute Kidney Injury (AKI), deep vein thrombosis (DVT), ventilatory days, ICU length of stay, hospital length of stay and in-hospital mortality.

The proposed Torode and Zieg system for pediatric pelvic fractures was used to classify each pelvic fracture pattern which categorizes pelvic fractures into four main types [17]. Type I represents fractures of bony prominences in avulsion; type II shows iliac crest fractures; type III corresponds to simple fractures of the pelvic ring without instability and type IV are complex fractures of the pelvic ring with instability. Grade IV is the most common indication of surgery.

Common types of surgical fixation of pediatric pelvic/acetabulum fractures include (1) ORIF of pelvis (plating symphysis pubic, plating of iliac bone)—For Torode I & II if there displacement more than 2–3 cm). (2) ORIF acetabulum—for comminuted fracture with displacement or articular step off more than 2 mm or in presence of intraarticular fragments, open fracture and in cases of central hip dislocations. (3) Pelvic external fixation to reduce pelvic volume to treat hemodynamic instability status. (4) Pinning or screw fixation of sacroiliac joint if there is widening/diastasis.

Ethical approval for this study was obtained from Research Ethics Committee, Medical Research Center at Hamad Medical Corporation (HMC) (IRB# 16395/16).

**Statistical analysis**: descriptive analyses were reported as frequencies and percentages for categorical variables. Continuous variables’ central tendency was described using means and standard deviations for variables with normal distribution and median and range for variables with non-normal distribution. Data were analyzed according to Torode classification for pelvic ring fracture (Type I–IV), open versus closed triradiate cartilage, and surgical versus non-surgical management. The continuous variables were analyzed using Student’s t-test and one-way ANOVA, as appropriate. Yates’ corrected chi-square was used for categorical variables, if the expected cell frequencies were below 5. For skewed continuous data non-parametric Mann-Whitney test was performed. A two-tailed *p* value of <0.05 was considered statistically significant. Data analysis was carried out using the Statistical Package for the Social Sciences, version 21 (SPSS, Inc., Chicago, IL).

## Results

**Fig. 1:**
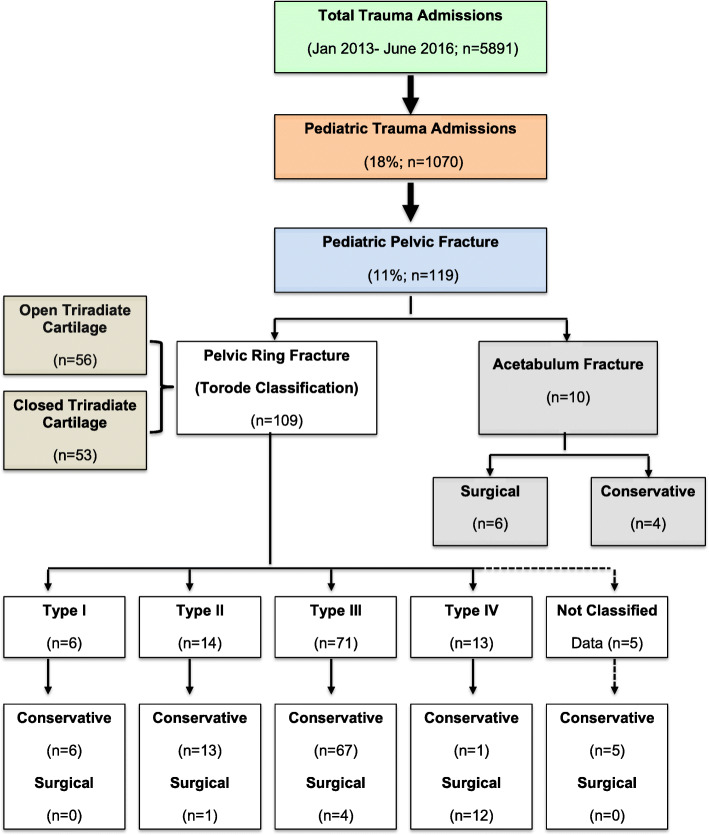
The study design (diagnosis and management)

During the study period, there were 5891 trauma admissions; of them 1070 (18%) were pediatric patients (≤18 years old). Among the pediatric admissions, there were 119 (11%) who had pelvic fractures. Figure 1 shows the study design (diagnosis and management). The majority were pelvic ring fractures (*n* = 109; 91.6%) while only 10 patients (8.4%) had an acetabular fracture. The mean age of patients was 11.5 ± 5.7 years, and the majority were males (78.2%) (Table 1). MVC was the mechanism of injury in half of the patients (52.9%), followed by pedestrian hit by car in a quarter (23.5%) and fall from height (12.6%). The most frequently associated injured body region was the chest (46.2%) followed by the abdomen (34.5%), spine (26.9%), head (24.4%), and lower extremity (17.6%).

**Table 1:** Demographics, clinical presentation and outcome of pediatric pelvic fracture based on management

	Overall *n* = 119	Conservative (*n* = 96; 80.7%)	Surgery (*n* = 23; *n* = 19.3%)	*P* value
**Age in years (mean ± SD**	11.5 ± 5.8	11.1 ± 5.8	13.5 ± 5.5	0.06
**Males %**	93 (78.2%)	75 (78.1%)	18 (78.3%)	0.98
**Mechanism of injury %**				
Motor vehicle crashes	63 (52.9%)	46 (47.9%)	17 (73.9%)	0.17 for all
Fall	15 (12.6%)	13 (13.5%)	2 (8.7%)	
Pedestrian	28 (23.5%)	24 (25.0%)	4 (17.4%)	
Others	13 (10.9%)	13 (13.6%)	0 (0.0%)	
**Associated injuries %**				
Chest	55 (46.2%)	49 (51.0%)	6 (26.1%)	0.03
Spine	32 (26.9%)	26 (27.1%)	6 (26.1%)	0.92
Abdomen	41 (34.5%)	34 (35.4%)	7 (30.4%)	0.65
Head	29 (24.4%)	26 (27.1%)	3 (13.0%)	0.15
Lower extremity	21 (17.6%)	17 (17.7%)	4 (17.4%)	0.97
**Injury Severity Score (mean ± SD)**	16.2 ± 11.0	15.9 ± 10.5	17.0 ± 13.3	0.68
**Revised Trauma Score (mean ± SD)**	7.3 ± 1.2	7.3 ± 1.2	7.6 ± 0.8	0.14
**Pelvis AIS**	2.2 ± 0.6	2.1 ± 0.4	2.7 ± 0.9	0.001
**Torode fracture classification % (*n*** **= 104)**				
Type I	6 (5.8%)	6 (6.9%)	0 (0.0%)	0.001 for all
Type II	14 (13.5%)	13 (14.9%)	1 (5.9%)	
Type III	71 (68.3%)	67 (77.0%)	4 (23.5%)	
Type IV	13 (12.5%)	1 (1.1%)	12 (70.6%)	
**Acetabular fracture %**	10 (8.4%)	4 (4.2%)	6 (26.1%)	0.001
**Open triradiate cartilage %**	56 (51.4%)	49 (56.3%)	7 (31.8%)	0.04 for all
**Closed triradiate cartilage %**	53 (48.6%)	38 (43.7%)	15 (68.2%)	
**Intubation %**	24 (20.2%)	21 (21.9%)	3 (13.0%)	0.34
**FAST scan % (n = 109)**	12 (11.0%)	8 (9.2%)	4 (18.2%)	0.22
**Blood transfusion %**	47 (39.5%)	34 (35.4%)	13 (56.5%)	0.06
**Number of blood units (median and range)**	3 (1–51)	4 (1–29)	2 (1–51)	0.52
**Massive blood transfusion %**	8 (6.7%)	5 (5.2%)	3 (13.0%)	0.17
**In-hospital complications %**				
Pneumonia	8 (6.7%)	7 (7.3%)	1 (4.3%)	0.61
Sepsis	2 (1.7%)	1 (1.0%)	1 (4.3%)	0.26
ARDS	1 (0.8%)	0 (0.0%)	1 (4.3%)	0.43
Acute Kidney Injury	4 (3.4%)	3 (3.1%)	1 (4.3%)	0.77
Deep vein thrombosis	1 (0.8%)	1 (1.0%)	0 (0.0%)	0.62
**Ventilatory days (median and range)**	7 (1–31)	6 (1–31)	7 (3–27)	0.60
**ICU length of stay (median and range)**	4 (1–35)	4 (1–33)	4.5 (1–35)	0.93
**Hospital length of stay (median and range)**	10 (1–85)	9 (1–85)	13 (1–74)	0.17
**Mortality %**	3 (2.5%)	2 (2.1%)	1 (4.3%)	0.53

Only 7 (5.9%) patients had pelvic hematoma with pelvic fracture, and the FAST was positive in 11% cases. The mean ISS was 16.2 ± 11, RTS was 7.3 ± 1.2 and pelvic AIS was 2.2 ± 0.5. The majority of patient were stable and were admitted to the floor (68; 57.1%), whereas 21 (17.6%) patients were transferred directly to operating room and 28 (23.5%) were admitted to ICU. The median ICU stay was 4 days (1–35) and the hospital length of stay was 10 days (1–85).

Table 1 compares the demographics, clinical presentation and outcome of pelvic fracture based on management. The two groups were comparable for demographics, mechanism of injury, injury severity and associated injuries except for chest injuries which were significantly higher in the conservatively treated group (51.0% vs. 26.1%, *p* = 0.03) than the surgical group. Patients in the surgical group were more likely to have higher pelvis AIS (*p* = 0.001), type IV fractures (*p* = 0.001), acetabular fractures (*p* = 0.001) and closed triradiate cartilage (*p* = 0.04) as compared to conservative group. On the other hand, type III fractures and open triradiate cartilage were significantly higher in the conservative group (*p* < 0.05). However, the rate of in-hospital complications and mortality did not differ significantly among the two groups.

Table 2 shows the clinical characteristics and outcome by types of pelvic ring fractures (Torode classification). Out of 109 pelvic ring fractures, 104 were classified and 5 were unclassified. The most common was type III (68.3%) followed by type II (13.5%), type IV (12.5%) and type I (5.8%). The distribution of age, gender, mechanism of injury, associated injuries, injury severity scores and presence of open or closed triradiate cartilage. Patients with type IV fractures were more likely to have mean higher pelvis AIS (*p* = 0.001) and surgical intervention (*p* = 0.001) as compared to other groups. Whereas, most lower grade fractures were treated conservatively (Fig. 1).

**Table 2:** Comparison of clinical presentation and outcome of paediatric pelvic fracture based on Torode fracture classification

	Type I (*n* = 6)	Type II (*n* = 14)	Type III (*n* = 71)	Type IV (*n* = 13)	*P* value
**Age (mean ± SD)**	10.4 ± 6.4	9.0 ± 6.7	11.1 ± 5.7	13.3 ± 5.3	0.30
**Males %**	4 (66.7%)	10 (71.4%)	55 (77.5%)	10 (76.9%)	0.91
**Associated injuries %**					
Chest	4 (66.7%)	7 (50.0%)	35 (49.3%)	5 (38.5%)	0.72
Spine	1 (16.7%)	1 (7.1%)	18 (25.4%)	4 (30.8%)	0.42
Abdomen	3 (50.0%)	4 (28.6%)	27 (38.0%)	6 (46.2%)	0.74
Head	2 (33.3%)	3 (21.4%)	20 (28.2%)	1 (7.7%)	0.42
Lower extremity	2 (33.3%)	2 (14.3%)	11 (15.5%)	2 (15.4%)	0.71
**Injury Severity Score (mean ± SD)**	21.0 ± 9.5	14.1 ± 8.7	15.7 ± 11.0	21.9 ± 15.8	0.19
**Revised Trauma Score**	7.8 ± 0.1	7.0 ± 1.3	7.2 ± 1.3	7.7 ± 0.4	0.30
**Pelvis AIS**	2.2 ± 0.4	2.1 ± 0.3	2.2 ± 0.4	2.9 ± 1.1	0.001
**Open triradiate cartilage %**	1 (33.3%)	10 (71.4%)	41 (58.6%)	4 (36.4%)	0.27
**Closed triradiate cartilage %**	2 (66.7%)	4 (28.6%)	29 (41.4%)	7 (63.6%)	
**Management %**					
Conservative management	6 (100%)	13 (92.9%)	67 (94.4%)	1 (7.7%)	0.001 for all
Surgical intervention	0 (0.0%)	1 (7.1%)	4 (5.6%)	12 (92.3%)	
**Blood transfusion**	3 (50.0%)	6 (42.9%)	26 (36.6%)	7 (53.8%)	0.64
**Massive blood transfusion**	0 (0.0%)	1 (7.1%)	4 (5.6%)	2 (15.4%)	0.54
**In-hospital complications %**					
Pneumonia	0 (0.0%)	1 (7.1%)	6 (8.5%)	0 (0.0%)	0.63
Sepsis	0 (0.0%)	0 (0.0%)	2 (2.8%)	0 (0.0%)	0.81
ARDS	0 (0.0%)	0 (0.0%)	0 (0.0%)	1 (7.7%)	0.07
Acute Kidney Injury	0 (0.0%)	1 (7.1%)	2 (2.8%)	1 (7.7%)	0.71
Deep vein thrombosis	0	0	1 (1.4%)	0	–
**ICU length of stay (median and range)**	2.5 (2–3)	5 (2–12)	5 (1–35)	4 (1–7)	0.44
**Hospital length of stay (median and range)**	13.5 (1–40)	5 (1–36)	9 (1–85)	13 (1–44)	0.15
**Mortality %**	0 (0.0%)	1 (7.1%)	1 (1.4%)	1 (7.7%)	0.43

The need for massive blood transfusion, in-hospital complications and mortality tended to be more in patients with higher facture grades (type III & IV) but did not reach statistical significance (Table 2).

**Acetabulum fractures:** Ten patients had acetabular fractures and were males, with mean age of 15.3 ± 1.4 years. They frequently involved in MVC and had more spinal and extremities associated injuries and half of them received blood transfusion. The mean ISS of patients with acetabulum fracture was lower than those with pelvic ring fracture (12.8 ± 5.4 vs. 16.5 ± 11.4; *p* = 0.09). Also, patients with acetabular fracture had shorter hospital course which may be due to better bony support for ambulation when compared to the immature paediatric pelvis and none of them developed in-hospital complications as compared to those with pelvic ring fracture.

**Fig. 2:**
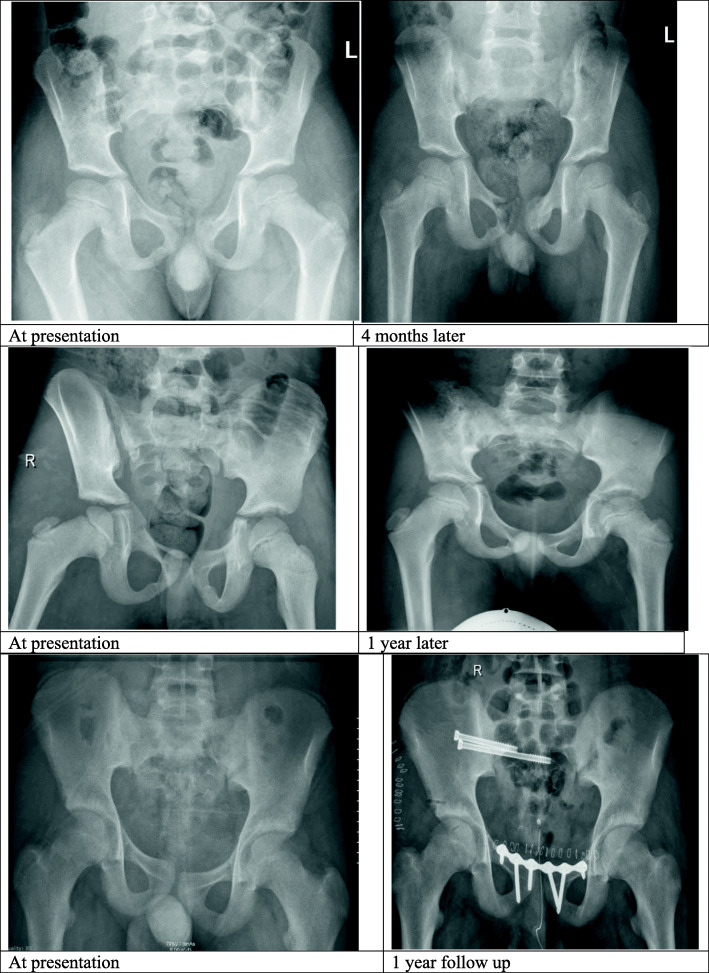
Hip X-ray before and after treatment of pediatric pelvic fractures: the upper panel shows Torode type III, treated non-operatively by non-weight bearing for 6 years old patient. The middle panel shows Torode type IV, treated by skeletal traction in 8 years old patient. The lower panel shows Torode type IV, treated by open reduction and internal fixation in 14 years old patient

**Open versus closed triradiate**: Comparison of clinical characteristics and outcomes of patients with open versus closed triradiate are shown in Table 3 and Fig. 1. There were 56 (51.4%) patients with open triradiate cartilage and 53 (48.6%) had closed triradiate cartilage. The closed group was significantly older in age (*p* = 0.001), predominantly males (*p* = 0.04) involved in MVC. Pedestrian hit by motor vehicle was the most common injury mechanism in the open group (*p* = 0.001). Also, patients with closed triradiate cartilage frequently sustained spine (*p* = 0.001) and head (*p* = 0.01) injuries, acetabular fracture (*p* = 0.001) and had higher mean ISS (*p* = 0.002) and pelvis AIS (*p* = 0.03) than the open group. The two groups were comparable for pelvic fracture classification, rate of intubation, massive blood transfusion, in-hospital complications and mortality. Patients with open triradiate cartilage were more likely to be treated conservatively whereas, closed group had significantly higher rate of surgical intervention (*p* = 0.04). The need for blood transfusion (*p* = 0.005), ventilatory days (*p* = 0.03), length of ICU (*p* = 0.005) and hospital stay (*p* = 0.001) were significantly higher in patients with closed triradiate cartilage compared to the open group. Figure 2 shows examples of hip X-ray after non-operative treatment, skeletal traction and surgically treated pediatric pelvic fractures.

**Table 3:** Demographics, and clinical presentation according to triradiate cartilage fractures (n = 109)

	Open triradiate cartilage (*n* = 56)	Closed triradiate cartilage (*n* = 53)	*P* value
**Age (mean ± SD)**	6.9 ± 4.2	16.4 ± 1.6	0.001
**Males %**	40 (71.4%)	46 (86.8%)	0.04
**Mechanism of injury %**			
Motor vehicle crashes	16 (28.6%)	41 (77.4%)	0.001 for all
Fall	9 (16.1%)	5 (9.4%)	
Pedestrian	25 (44.6%)	1 (1.9%)	
Others	6 (10.7%)	6 (11.3%)	
**Associated injuries %**			
Chest	22 (39.3%)	26 (49.1%)	0.30
Spine	4 (7.1%)	22 (41.5%)	0.001
Abdomen	16 (28.6%)	24 (45.3%)	0.07
Head	8 (14.3%)	18 (34.0%)	0.01
Lower extremity	7 (12.5%)	13 (24.5%)	0.10
**Injury Severity Score (mean ± SD)**	12.8 ± 9.9	19.3 ± 11.6	0.002
**Revised Trauma Score (mean ± SD)**	7.6 ± 0.9	7.2 ± 1.3	0.06
**Pelvis abbreviated injury score (mean ± SD)**	2.1 ± 0.4	2.4 ± 0.7	0.03
**Torode fracture classification %**			
Type I	1 (1.8%)	2 (4.8%)	0.27 for all
Type II	10 (17.9%)	4 (9.5%)	
Type III	41 (73.2%)	29 (69.0%)	
Type IV	4 (7.1%)	7 (16.7%)	
**Acetabular fracture %**	0 (0.0%)	9 (17.0%)	0.001
**Management %**			
Conservative management	49 (87.5%)	38 (71.7%)	0.04
Surgical intervention	7 (12.5%)	15 (28.3%)	
**Intubation %**	8 (14.3%)	14 (26.4%)	0.11
**Positive FAST scan %**	4 (7.5%)	8 (17.0%)	0.14
**Number of blood units (median and range)**	2 (1–12)	4 (1–51)	0.06
**Blood transfusion %**	15 (26.8%)	28 (52.8%)	0.005
**Massive blood transfusion %**	2 (3.6%)	5 (9.4%)	0.21
**In-hospital complications %**			
Pneumonia	0 (0.0%)	6 (11.3%)	0.10
Sepsis	0 (0.0%)	2 (3.8%)	0.14
ARDS	0 (0.0%)	1 (1.9%)	0.30
Acute Kidney Injury	1 (1.8%)	3 (5.7%)	0.28
**Ventilatory days (median and range)**	2 (1–10)	9 (1–31)	0.03
**ICU length of stay (median and range)**	2.5 (1–15)	7 (2–35)	0.005
**Hospital length of stay (median and range)**	6 (1–76)	14 (1–85)	0.001
**Mortality %**	1 (1.8%)	2 (3.8%)	0.52

Table 4 shows the type of surgical intervention based of the fracture classification.

**Table 4:** Management of pelvic fracture

Torode classification and acetabulum Fracture	Open/Closed triradiate	Type of surgery
III	Closed	Left Sacroiliac screws
IV	Open	k-wire fixation of Sacroiliac joint
Acetabular fracture	Closed	Total Hip Replacement/acetabulum
IV	Open	k-wire fixation of Sacroiliac joint/ORIF
IV	Closed	Plating of iliac bone
IV	Closed	Sacroiliac screw
Acetabular fracture	Closed	Screw fixation
Acetabular fracture	Closed	Acetabulum
IV	Closed	Left Sacroiliac screw
IV	Closed	Left Sacroiliac screw
IV	n/a	Pelvis and acetabulum ORIF (done abroad)
III	Closed	Pelvis not fixed (only Right femur/hip DHS)
IV	Open	Skeletal traction
IV	Closed	ORIF, Sacroiliac screw, Symphysis pubis plating
III	Open	Pelvis not fixed (only femur hip spica application)
Acetabular fracture	Closed	Examination under anesthesia for right hip stability
Acetabular fracture	Closed	ORIF
IV	Open	skeletal traction
III	Open	Treated non operatively
IV	Closed	ORIF
Acetabular fracture	Closed	Examination under anesthesia for left hip stability
IV	Closed	Skeletal traction
II	Open	Treated non operatively (only ORIF for left femur subtrochanteric fracture)

*DHS* dynamic hip screw, *ORIF* open reduction internal fixation, *n/a* not available

## Discussion

This is a single center study that describes a 3 and half year experience for the prevalence, clinical characteristics, injury pattern and management of PPF at the only level I trauma center in Qatar. Pelvic fracture in the pediatric population is uncommon which corresponds to 2.3% of the total trauma admissions in our centre. The key findings of this study include the predominance of male gender, MVC as major mechanism of injury; chest and abdomen were the frequently associated injured body regions. In addition, patients who required surgical intervention sustained severer pelvic injuries with higher pelvis AIS, frequent type IV fractures, acetabular fractures and closed triradiate cartilage. However, the rate of in-hospital complications and mortality did not differ significantly with respect to management, type of fracture and open or closed triradiate cartilage.

The high-energy trauma includes MVC, pedestrian hit by a car and falling from height results in pelvic fracture of immature bone in children and is an indicator of the presence of associated injuries [18, 19]. The present study observed traffic accidents as the most common mechanism of injury which is similar to that of the adult pelvic fracture patients [20]; a finding that was reported by several earlier studies [4, 9, 10, 18, 19].

Despite the similar injury mechanism causing pelvic fracture in adults, the pattern of fracture among pediatric patients seems to be more stable [14, 18]. Therefore, the clinically useful classification could facilitate the prediction of the disease process’s natural history and guide in selecting the appropriate management strategy. Torode and Zieg classification [17] is the widely used system for pediatric pelvic fractures. This system attributes both anatomic and mechanical factors to the severity of the deformity. However, it did not consider the bony pelvis’ changing maturity throughout the children’s age range. In our cohort, Torode type III was the most common pelvic ring injury (68.3%) which is similar to what has been reported previously [6, 8, 18]. On the other hand, Niedzielki et al. [21] reported that the frequent type of fracture among children is avulsion fractures secondary to sports injuries. The fracture classification influences the management plan for fracture and predicts length of hospital stay and outcomes. In our study, patients with type IV fractures were more likely to undergo surgical intervention.

Associated pelvic hematoma is less common in children when compared to adults and more easily controlled [12]. In our cohort, only 7 patients were identified to have pelvic hematoma. Rapid fatal exsanguination due to pelvic fracture is extremely rare in children, while it is the leading and common cause for early mortality in adult polytrauma patients. This difference may be due to the vasoactive proprieties of pediatric blood vessels that undergoes more vasoconstriction as compared to the more friable atherosclerotic adult vessels [12, 22]. Around 17–46% of pediatrics trauma victims with pelvic fracture required blood transfusion. However, it appears that the indication for the transfusion is mainly to restore the RBC mass rather than the resuscitation of life-threatening hypovolemia and usually, there is no need for embolization to control the bleeding. Concomitant solid visceral injury causes bleeding and the incidence of intra-abdominal organ injury varied between 10 and 20%. Also, the pelvic hematoma is not a significant contributing factor for mortality [12, 14].

Earlier studies demonstrated an association between other injuries and pelvic fracture that may leads to early or late mortality, if not managed timely [4, 23]. The present study showed chest injury to be the most common associated injury followed by abdominal and head injuries in pediatric polytrauma patients. These findings are consistent with earlier report [4]. Whereas, others reported the head as frequently associated injury than thorax and abdomen [5, 23]. On the other hand, femoral and tibia fractures were the most common associated fractures in the limbs. In contrast, spine fracture, mainly thoracic spine, was the most common associated fracture in the axial skeleton [3, 5, 19] which is similar to our findings.

The 24-h mortality for pediatrics polytrauma patients is related to many factors, including the injury severity score; patients associated with higher ISS scores were hemodynamically unstable and required blood transfusion. Another major factor is the severity of the associated head injury. Death increased with the head AIS and other outcomes such as longer ICU stay. In our study the overall mortality is 2.5% and two out of three patients died due to concomitant head injury. Notably, the severity of pelvic ring injury was not associated with early mortality [12, 19, 24, 25]. The increase in the mortality rate among adult polytrauma patients has been correlated with increased patient age, while this correlation was not observed in pediatrics polytrauma patients [12].

During adolescence the pediatric pelvic transforms into adult form and during this transition, the pelvis primarily loses elasticity, and the triradiate cartilage closure occurs at approximately 14 years of age in males and 12 years in females. In our study, around half of patients had open triradiate cartilage and half had closed triradiate cartilage. The closed group was older in age, predominantly males and had higher mean ISS and pelvis AIS. The biological transition has been suggested to distinguish pediatric pelvis from the adults and is an essential factor which may influence the treatment choices as well as the outcome [6, 14, 26].

Earlier, most pelvic fracture cases were treated non-operatively due to the possibility of pelvic remodeling during the successive growth in children like the long bone fracture [27]. However, Conservative treatment of unstable and displaced pelvic fracture may result in pelvic asymmetry leading to more serious disabilities and chronic pelvic pain like that of adults.

In the present study, patients with type IV fractures were more likely to have surgical intervention while lower grade fractures were treated conservatively. Prevention of deformity after displacement of pelvic fracture is one of the indications for surgical intervention in pediatric patients, which leads to more effective rehabilitation and nursing care [2, 28, 29].

Limitations of this study include the retrospective design, and lack of follow-up (long-term) and quality of life data. The current study had less Torode type 1 avulsion injuries; this could be related to that those minor sports injuries were not admitted to the Level 1 trauma center. The present study is post-hoc analysis of traumatic pelvic injuries database that has been described previously [20]. Moreover, the pediatric age varied widely which potentially mix the biological differences between children and teenagers which tend to behave more like adults. Although maturation occurs earlier than the cut off in this report, we opted to the WHO definition of pediatric age cutoff. The time from injury to intervention was not captured in the database. We have included all patients with open as well as closed triradiate cartilage which differs from patient-to-patient depending on gender and bony growth. Open triradiate cartilage mostly were observed in younger children (mean age: 6.9 ± 4.2 years), whereas, adolescents (mean age: 16.4 ± 1.6 years) were more likely to have closed triradiate fractures in our cohort. The closure of the triradiate cartilage usually occurs at age of 15 in males and age of 13 in females [30].

## Conclusions

This study shows that PPF are uncommon and mainly caused by high-impact trauma associated with multisystem injuries. The majority of pediatric fracture pattern are stable, despite the underlying high-energy mechanism due to the high plasticity and elasticity of bones. Polytrauma is the usual pattern that dictates the outcome rather than the fractures. Management of PPF mainly depends on the severity of fracture as patients with higher grade fractures require surgical intervention. Also, associated injuries should be managed appropriately to minimize the morbidity and mortality. Furthermore, larger prospective study is needed to understand the age-related pattern and management of PPF.

## Data Availability

All data generated or analyzed during this study are included in this manuscript. De-identified data are accessible upon agreement with the national trauma registry and the medical research centre at Hamad Medical Corporation (contact: mrchelpdesk@hamad.qa).

## Abbreviations

AIS: Abbreviated injury scale
ISS: Injury severity score
PPF: Pediatric pelvic fractures
RTS: Revised trauma score
